# Efficacy of Thai Plant Extracts for Antibacterial and Anti-Biofilm Activities against Pathogenic Bacteria

**DOI:** 10.3390/antibiotics10121470

**Published:** 2021-11-30

**Authors:** Dennapa Saeloh, Monton Visutthi

**Affiliations:** 1 Faculty of Medical Technology, Prince of Songkla University, Hat Yai, Songkhla 90110, Thailand; dennapa.sa@psu.ac.th; 2 Biology Program, Faculty of Science and Technology, Nakhon Ratchasima Rajabhat University, Nakhon Ratchasima 30000, Thailand

**Keywords:** antibacterial, anti-biofilm, *Piper betle*, *Staphylococcus aureus*, *Escherichia coli*, Thai plant extracts

## Abstract

The emergence of drug-resistant bacteria has impacted the outcome of current therapeutics as a threat to global healthcare; novel medicines are urgently needed. Thirteen medicinal plants were collected in Northeastern Thailand, and their crude ethanolic extracts were evaluated for antibacterial activities against *Staphylococcus aureus* ATCC25923 and *Escherichia coli* ATCC25922 using the broth micro-dilution method. *Piper betle* leaf ethanolic extract showed optimal activity against both representative bacterial strains. Activity was also observed against clinical isolates of methicillin-resistant *S. aureus* (MRSA) and *E. coli*, with minimal inhibitory concentration (MIC) ranging from 0.31 mg/mL to 2.5 mg/mL and minimal bactericidal concentration (MBC) ranging from 0.62 mg/mL to 2.5 mg/mL. A time-kill study revealed that the extract activity was time- and dose-dependent, and also bactericidal on the tested bacteria. *P. betle* extract inhibited biofilm formation and promoted biofilm eradication in both *S. aureus* and *E. coli.* 4-Allyl-1,2-diacetoxybenzene and eugenol were identified as the most abundant compounds in the extract and may play major roles in the anti-bacterial and anti-biofilm activity. Results suggest that ethanolic *P. betle* leaf extract shows promise as an alternative method for the prevention of bacterial diseases.

## 1. Introduction

Drug-resistant bacteria are now a serious global healthcare problem, leading to poor outcomes of current therapeutics. Biofilm composed of polysaccharides, proteins, nucleic acids, lipids and other organic components is considered an essential bacterial virulence factor. Microorganisms generate biofilms and form a community on the host surface to protect themselves from the environment. Biofilms are mostly found in medical, industrial, food processing and water distribution systems [1,2]. Biofilm-embedded bacteria are able to increase their defenses against antibiotics at up to a thousand-fold, compared to planktonic cells [3]. Most current antibiotics are effective against unattached bacteria, but the treatment of biofilm infections requires antibiotics at high concentrations, usually above peak serum levels. Therefore, therapeutics for latent and recurrent infections are less effective [4]. The elimination of bacteria within the biofilm requires degradation of the biofilm matrix or the discovery of antibiotics that can enter through the biofilm.

Currently, natural products have attracted widespread interest in the search for alternative medicines [5,6,7,8,9]. Plants are excellent sources of antimicrobials that have minimal treatment side effects [5]. Thai herbal medicines have been used successfully to treat many bacterial infections [6,7,8]. The antibacterial activity of Thai plants is well documented but research concerning their anti-biofilm activity is sparse. Previous studies screened the antibacterial and anti-biofilm activity of Thai medicinal plant extracts against oral pathogens [9]. A source of antibacterial and anti-biofilm agents was determined to be 4-chromanol in an ethanolic extract of *Piper betle* leaves [9], illustrating the potential application of natural products in the therapeutic prevention of diseases associated with oral biofilms. However, investigation of the efficacy of Thai herbs in combatting foodborne and nosocomial pathogens, such as *Staphylococcus aureus* and *Escherichia coli*, has not been widely conducted. 

Here, the antibacterial and anti-biofilm activities of selected indigenous Thai plants, commonly used for bacterial infection against *S. aureus* and *E. coli,* were evaluated.

## 2. Results

### 2.1. Antibacterial Activity of Plant Extracts

Thirteen ethanolic extracts from selected plants were tested for antibacterial activity against Gram-positive and Gram-negative bacteria by the broth microdilution method. The primary results showed that *P. betle* leaf extract inhibited the reference bacteria (Table 1), and this leaf extract was selected as a representative agent to evaluate antibacterial activity against 91 clinical bacterial isolates. The results in Table 2 show the MICs of *P. betle* extract against Gram-positive and Gram-negative bacteria. The MIC and MBC values of the extract for hospital isolates of MRSA ranged from 0.31 mg/mL to 2.5 mg/mL and from 0.31 mg/mL to 5.0 mg/mL, respectively, while the MIC and MBC values of the extract against clinical isolates of *E. coli* were 1.25 mg/mL to 2.5 mg/mL and 2.5 mg/mL to 5.0 mg/mL, respectively.

### 2.2. Time-Kill Kinetic Assay of Piper betle Extract against the Bacteria

**Figure 1:**
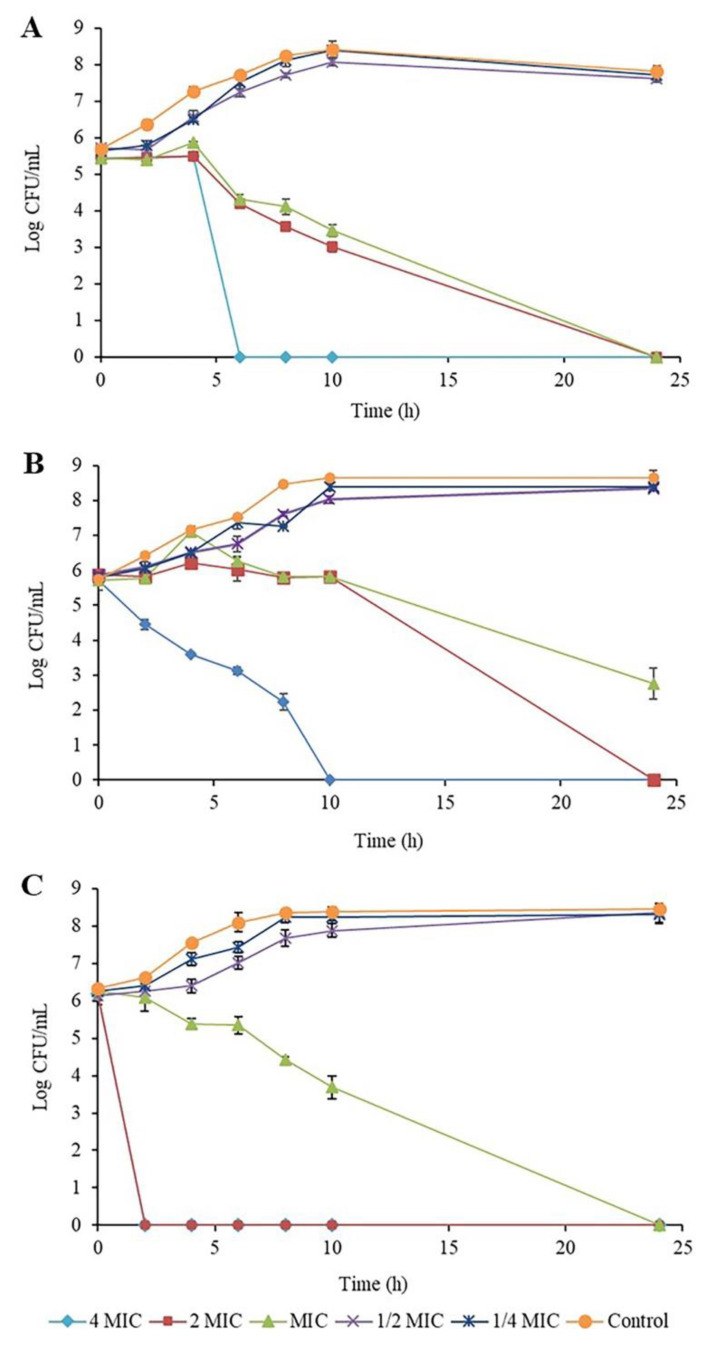
Time-kill determination of (**A**) *Staphylococcus aureus* ATCC29213, (**B**) methicillin-resistant *S. aureus* NPRC001R and (**C**) *Escherichia coli* ATCC25922 after treatment with *Piper betle* leaf ethanolic extract.

A time-kill kinetic assay of *P. betle* extract against the reference strains, *S. aureus* ATCC29213, MRSA NPRC001R and *E. coli* ATCC25922, was investigated, with the results demonstrated in Figure 1A–C, respectively. Patterns of cell survival and cell decrease after treatment with different concentrations of the extract were similar among different bacteria. A time-kill study revealed that the extract activity was time- and dose-dependent, while the extract also exhibited a bactericidal effect on the tested bacteria. At 2MIC and 4MIC, the extract reduced bacterial cells at the starting log CFU/mL by more than 3 logs within 24 h, with *E. coli* showing a decrease within 2 h. At MIC, the extract reduced bacterial cells within 24 h, except for MRSA, which decreased by more than 2 logs. These results were consistent with antibacterial activity. 

### 2.3. Biofilm Inhibitory and Eradicating Activity of Piper betle Extract

**Figure 2:**
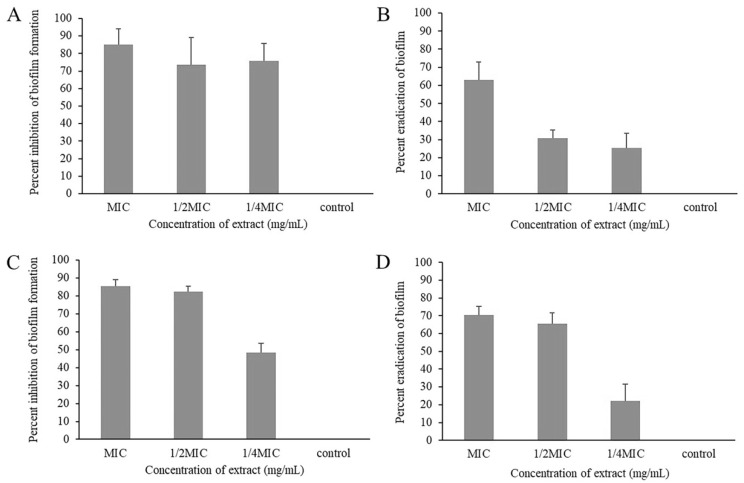
Inhibition of biofilm formation and biofilm eradication potential of *Piper betle* leaf ethanolic extract at various concentrations against (**A**,**B**) *Staphylococcus aureus* ATCC29213 and (**C**,**D**) *Escherichia coli* ATCC25922.

The effects of *P. betle* extract on the inhibition and eradication of biofilm formation against *S. aureus* ATCC29213 and *E. coli* ATCC25922 are demonstrated in Figure 2. Sub-inhibitory concentration (1/4MIC and 1/2MIC) and MIC of *P. betle* showed significant biofilm inhibiting activity against tested strains (*p* < 0.05) (Figure 2A,C). The inhibition ranged 70–85% with *S. aureus* and 50–85% with *E. coli*. The biofilm eradicating activity of the ethanolic extract is shown in Figure 2B,D. The biofilm removal capacity of the extract at 1/4MIC, 1/2MIC and MIC was 25%, 30%, and 60% for *S. aureus* and 20%, 65%, and 70% for *E. coli*.

### 2.4. Inhibition of Staphyloxanthin Biosynthesis in Staphylococcus aureus by Piper betle

**Figure 3:**
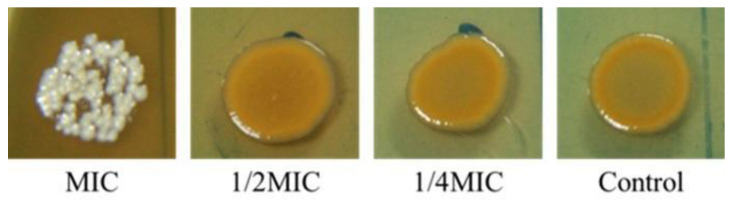
Pigmentation of *Staphylococcus aureus* ATCC29213 grown on TSA with or without *Piper betle* leaf ethanolic extract supplemented with various concentrations at 37 °C for 24 h.

The effect of *P. betle* extract on staphyloxanthin biosynthesis in colonies of *S. aureus* was evaluated. The extract exhibited complete inhibition of staphyloxanthin biosynthesis of the bacterial colonies at MIC value (Figure 3).

### 2.5. Phytochemical Analysis of Piper betle Leaf Extract

Thirty phytochemical components were identified from the ethanolic extract of *P. betle* leaves by GC and GC-MS analyses. Major constituents calculated from peak areas showed that 4-allyl-1,2-diacetoxybenzene (70.32%) and eugenol (18.80%) were recorded as the highest two amounts (Table 3).

## 3. Discussion

The emergence of drug-resistant bacteria has impacted the outcome of current therapeutics as a threat to global healthcare.Therefore, searching for an alternative treatment to solve this problem has attracted increasing interest. Over thousands of years, 80% of the global population has used traditional herbal medicines to treat various diseases [10,11]. Plants are a valuable source of medicines. Many demonstrate antibacterial efficacy for the treatment of bacterial infections. In this study, the antimicrobial and anti-biofilm activities of 13 plant extracts were tested against bacteria. All plants were obtained from Nakhon Ratchasima Province in Northeastern Thailand. Ethanol was used for plant extraction to obtain a high number of active agents with no toxicity [12]. *S. aureus*, MRSA and *E. coli* were selected as representatives of Gram-positive bacteria, antibiotic-resistant bacteria and Gram-negative bacteria, respectively. 

Differences in the MIC and MBC values of the crude ethanolic extracts demonstrated selective antibacterial activity. Four of the thirteen extracts, including *Boesenbergia rotunda*, *Caseria grewiifolia*, *P. betle* and *Rhodomyrtus tomentosa,* showed antibacterial activity against *S. aureus* and MRSA, with MIC values less than 1 mg/mL. These were considered active crude extracts [13]. The MIC index (MIC/MBC) for all extracts was less than four, indicating bactericidal effects on *S. aureus* and MRSA [14]. Moreover, the lowest MIC value was recorded on a standard strain of *E. coli*, while Gram-negative bacteria were found in *P. betle*. The antimicrobial effects were confirmed in more isolates with MIC ranges of less than 1 mg/mL. *P. betle* presented bactericidal potential with a broad spectrum. Time-kill determination demonstrated that the antibacterial efficacy of the extract on Gram-negative bacteria was faster than for Gram-positive bacteria. Furthermore, *P. betle* possessed strong anti-biofilm activity with dual actions of preventing and eradicating the biofilm of *S. aureus* and *E. coli*. In addition, *P. betle* also inhibited staphyloxanthin, the golden pigment of *S. aureus*. Although the biofilm cells produced staphyloxanthin at higher levels than the planktonic cells [15], the extract obviously inhibited pigment production of biofilms, possibly resulting from the loss of the biofilm-forming ability in the bacteria. 

An earlier study recorded that the ethanolic leaf extract of *P. betle* mainly contained 4-chromanol with antibacterial and anti-biofilm activity against oral pathogens [9]. By contrast, here, 4-allyl-1,2-diacetoxybenzene was identified as the most dominant compound. Despite using the same solvent to extract bioactive compounds from the plant, the varying extraction methods and conditions provided different chemical compounds [16]. 4-Allyl-1,2-diacetoxybenzene might play a major role in significant antibacterial activity. This idea concurred with previous reports indicating that essential oils of *Melaleuca* species rich in 4-allyl-1,2-diacetoxybenzene exhibited bacteriostatic and bactericidal effects against foodborne pathogens [17,18]. In this study, eugenol was the second most abundant compound in *P. betle* leaf extract. Eugenol was detected as a major component in essential oils from *Etlingera sayapensis*, which was characterized as being highly effective against a variety of Gram-positive and Gram-negative bacteria [19]. Correspondingly, the remarkable antibacterial activity of clove and cinnamon essential oil were attributed to the eugenol content [20,21]. Moreover, typical biofilm and cell membrane structures were shown to be disturbed by eugenol [22]. 

## 4. Materials and Methods

### 4.1. Plant Materials and Extract Preparation

Thirteen plant species were sourced from Nakhon Ratchasima Province, Thailand with details shown in Table 1. Each plant material was dried at 60 °C for 3–5 days, powdered and extracted by soaking in 95% ethanol for one week and then filtrated through Whatman No. 4 filter paper. Each filtrate was evaporated under reduced pressure in a rotary evaporator until complete dryness and kept at 4 °C until required for antibacterial testing.

### 4.2. Bacterial Strains and Growth Conditions

*S. aureus* ATCC29213, MRSA NPRC001R and *E. coli* ATCC25922 were obtained from the Division of Biological Science and Natural Products Research Center, Faculty of Science, Prince of Songkla University. In total, 48 isolates of MRSA were obtained from the Songklanagarind Hospital, and 43 isolates of *E. coli* were received from Maharat Nakhon Ratchasima Hospital as clinical specimens. The bacteria were maintained on tryptic soy agar (TSA) and strains were grown under aerobic condition (37 °C, 16–18 h).

### 4.3. Evaluation of Antibacterial Activity

#### 4.3.1. Determination of Minimum Inhibitory Concentrations (MICs)

The broth microdilution method was carried out according to the CLSI guideline [23]. The extracts were serially diluted two-fold in Mueller Hinton broth (MHB) in 96-well microtiter plates to obtain final concentrations ranging from 0.15 mg/mL to 10 mg/mL. Then, an equal volume of 100 µL of log-phase bacterial culture, approximately 10^6^ CFU/mL, was added to each well. After incubation at 37 °C for 16–18 h, bacterial growth was observed by the turbidity of the medium. The MIC was considered the lowest concentration of the extracts or antibiotics required to inhibit bacterial growth without turbidity of the medium compared with the negative control. All assays were performed in triplicate.

#### 4.3.2. Determination of Minimum Bactericidal Concentrations (MBCs) 

All wells showing bacteria inhibited in the broth microdilution method were plated on Mueller Hinton agar (MHA) with 10 µL aliquots of the contents and grown at 37 °C for 16–18 h. The MBC was recorded as the lowest concentration of the extracts or antibiotics that killed 99.9% of bacteria, showing no growth on MHA. All assays were performed in triplicate.

#### 4.3.3. Time-Kill Assay

The primary results of the plant extracts revealed that *P. betle* leaf extract showed the strongest antibacterial activity on *S. aureus* and *E. coli*. The antibacterial activities of *P. betle* leaf extract on *S. aureus*, MRSA and *E. coli* were studied using a time-kill assay. A bacterial culture (5·10^5^ CFU/mL) was added to MHB containing the extract at 4MIC, 2MIC, MIC 1/2MIC and 1/4MIC, and untreated cultures were incubated at 37 °C. The samples were collected at 0, 2, 4, 6, 8, 10, 12 and 24 h by culturing on TSA plates. A control incubation was performed with 1% DMSO. Surviving colony bacteria were counted, and log10 CFU/mL was calculated. A time-kill curve was analyzed by plotting log CFU/mL against time.

### 4.4. Evaluation of Anti-Biofilm Activity

#### 4.4.1. Biofilm Formation

The effect of *P. betle* extract on the biofilm formation of each representative strain of *S. aureus* and *E. coli* was examined, using the modified microdilution method [24]. Briefly, each bacterial strain was grown in tryptic soy broth (TSB) containing 1% glucose overnight. The culture was diluted to 10^6^ CFU/mL and transferred to a 96-well microtiter plate containing two-fold serial dilutions of *P. betle* extract at 4MIC, 2MIC, MIC, 1/2MIC and 1/4MIC. After incubation at 37 °C for 24 h, each well of microtiter plate was washed twice with phosphate buffer saline (PBS), fixed by absolute methanol for 20 min, and dried overnight. After that, the wells were stained with 200 µL of 2% crystal violet solution for 15 min. Subsequently, the plate was washed with water and air-dried. Stained biofilms were dissolved in 200 µL of 33% (*v*/*v*) glacial acetic acid, and the plate was measured at 570 nm using a microplate reader (BioTek, Winooski, VT, USA). The relative percentage of biofilm formation was defined as (mean 570 nm of treated well/mean 570 nm of control well) × 100.

#### 4.4.2. Biofilm Eradication

Established biofilms were cultured as defined by Saising et al. [25]. Two hundred microliters of growing culture (10^6^ CFU/mL) were transferred to a 96-well microtiter plate and incubated at 37 °C for 24 h. Then, planktonic cells were removed and 100 µL of TSB was added. After that, *P. betle* extract at different concentrations was added. After incubation at 37 °C for 24 h, each well of the microtiter plate was washed with PBS twice and air-dried. The plate was then used for biofilm formation.

#### 4.4.3. Production of Staphyloxanthin

The ability of *P. betle* extract to reduce the production of the golden yellow pigment staphyloxanthin was investigated. An overnight culture of the tested strains in TSB was prepared. The bacterial suspensions were diluted 1:100 in a TSA plate containing MIC, 1/2MIC and 1/4MIC of *P. betle* extract. A control plate without the extract was prepared in the same way. The cultural plates were incubated for 24 h at 37 °C. The pigment colonies were photographed, and the golden yellow pigment was compared in treated and untreated samples.

### 4.5. Bioactive Compound Assay

Gas chromatography-mass spectrometry (GC-MS) analysis of ethanolic extracts of *P. betle* leaves was conducted at the Center for Scientific and Technological Equipment, Suranaree University of Technology, Thailand. GC-MS was performed by a Bruker series 3XO model with an electron ionization detector (Karlsruhe, Germany) operated through a data system. One microliter of extract (50 mg/mL) was added to the injection port of the GC column. The identification of individual compounds was made by mass spectra library matching against the database of National Institute Standard and Technology (NIST MS 14.0). The matching result for a compound was represented by percent of probability values (%Prob). Relative percentages of the chemical compositions were calculated by considering the summation of all GC peak areas in the total ion chromatogram of one sample as 100%. Each peak percentage area (%Area) was obtained by dividing its area by the total area of all compounds.

### 4.6. Statistical Analysis

Data were expressed as the mean and standard deviation (SD) by computational analysis from the three experiments, with duplicate or triplicate independent experiments.

## 5. Conclusions

*P. betle* has high antibacterial and anti-biofilm potential as a promising anti-infective phytotherapeutical. This natural agent presents positive options which are appropriate alternative approaches to control pathogenic bacteria. The ability of *P. betle* extract to prevent *S. aureus* and *E. coli*, especially in the case of biofilms, may be beneficial for the food industry and medical devices where these pathogens are generally found.

## Figures and Tables

**Table 1:** Minimum inhibitory concentration (MIC) and minimum bactericidal concentration (MBC) of selected plant extracts against pathogenic bacteria.

Medicinal Plant	Common Name	Local Name	Plant Part	Susceptibility Testing (mg/mL)		
				*S. aureus* ATCC29213	MRSA NPRC001R	*E. coli* ATCC25922
*Artocarpus lakoocha* Roxb.	Green Tampang	Ka noon pa	Leaf	10/10	10/10	10/>10
*Boesenbergia rotunda* (Roxb.) Schltr.	Fingerroot	Krachai	Rhizome	0.15/0.15	0.15/0.15	>10/>10
*Casearia grewiifolia* Vent.	-	Kruai Pa	Fruit	0.62/0.62	1.25/2.5	>10/>10
*Chromolaena odorata* (L.) R.M.King & H. Rob.	Christmas bush	Sap suea	leaf	5/>10	10/>10	>10/>10
*Limnophila aromatica* (Lam.) Merr	Rice paddy herb	Phak kha Yeang	Whole plant	2.5/5	2.5/5	>10/>10
*Millingtonia hortensis* Linn.	Cork tree	Hian	Leaf	2.5/>2.5	2.5/2.5	5/>10
*Oroxylum indicum* (L.) Kurz.	Broken bones plant	Pheka	Leaf	2.5/>2.5	2.5/>2.5	>10/>10
*Piper betle* Linn.	Betel	Plue	Leaf	0.62/0.62	0.62/0.62	2.5/2.5
*Rhodomyrtus tomentosa* (Aiton) Hassk.	Rose myrtle	Phruat	Leaf	0.31/0.62	0.62/0.62	>10/>10
*Syzygium cumini* Linn.	Black plum	Waa	Leaf	2.5/5	2.5/5	10/>10
*Xanthostemon chrysanthus* (F.Muell.) Benth.	Golden myrtle	Rak raek pop	Leaf	1.25/2.50	1.25/2.50	>10/>10
*Zingiber officinale* Roscoe.	Ginger	Khing daeng	Rhizome	1.25/>10	>10/>10	10/>10
*Ziziphus mauritiana* Lam.	Jujube	Phutsa	Leaf	2.5/10	5/>10	>10/>10

**Table 2:** MIC and MBC values of *Piper betle* ethanolic extract against other isolates of methicillin-resistant *Staphylococcus aureus* (MRSA) and *Escherichia coli*.

Number of Isolates	Antibacterial Activities of *Piper betle* Ethanol Extract	
	MIC Range (mg/mL)	MBC Range (mg/mL)
MRSA (*n* = 48)	0.31–2.5	0.31–5.0
*E. coli* (*n* = 43)	1.25–2.5	1.25–5.0

**Table 3:** Chemical constituents of *Piper betle* leaf ethanolic extract.

No.	RT (min)	CAS RN	Extract Constituent	%Area	%Prob
1	10.841	501-92-8	4-Allylphenol	0.33	71.0
2	15.358	97-53-0	Eugenol	18.80	27.4
3	16.891	87-44-5	Caryophyllene	0.90	31.7
4	18.162	6753-98-6	Alpha-caryophyllene	0.25	49.1
5	19.086	1460-97-5	Gamma-cadinene	1.21	29.1
6	20.371	13620-82-1	4-Allyl-1,2-diacetoxybenzene	70.32	42.2
7	20.938	483-76-1	Delta-cadinene	0.78	33.6
8	23.129	6750-60-3	(-)-Spathulenol	0.06	29.7
9	23.302	1139-30-6	Caryophyllene oxide	0.07	27.4
10	36.594	628-97-7	Hexadecanoic acid, ethyl ester	0.15	45.1
11	39.109	150-86-7	Phytol	0.76	83.5
12	39.992	544-35-4	Linoleic acid ethyl ester	0.15	20.8
13	40.114	112-63-0	Linoleic acid methyl ester	0.23	13.9
14	41.012	10236-16-5	Phytol acetate	0.76	51.4
15	42.057	3033-62-3	Bis(2-(dimethylamino)ethyl) ether	0.16	39.2
16	43.470	122-79-2	Acetic acid, 3-(adamant-2-ylidene-methoxymethyl)-,phenyl ester	0.10	57.8
17	44.000	1778-02-5	6,16-Dimethyl-20-oxo pregn-5-en-3-yl acetate	0.13	23.2
18	44.403	604-09-1	14-Hydroxypregn-4-ene-3,20-dione	0.17	22.0
19	45.428	23470-00-0	Glycerol beta-palmitate	0.69	65.0
20	45.575	542-44-9	Glycerol alpha-palmitate	0.07	41.0
21	46.785	55268-70-7	Hexadecanoic acid, 2,3-bis(acetyloxy)propyl ester	0.24	59.8
22	47.785	3443-82-1	Beta-monolinolein	0.42	27.7
23	47.870	56797-43-4	Cis,cis,cis-7,10,13-hexadecatrienal	0.31	29.4
24	48.976	55401-63-3	9-Octadecenoic acid (*Z*)-, 2-(acetyloxy)-1-[(acetyloxy)methyl] ethyl ester	0.22	43.5
25	53.759	7695-91-2	DL-alpha-tocopherol	0.21	53.7
26	55.666	474-62-4	Campesterol	0.30	30.2
27	56.364	83-48-7	Stigmasterol	0.35	43.4
28	57.809	83-47-6	Gamma-sitosterol	1.15	75.0
29	59.655	1259-10-5	Cycloartenol acetate	0.20	7.3
30	61.465	79897-80-6	Stigmastan-3,5-diene	0.51	66.0

## Data Availability

Not applicable.
